# The economic burden of advanced liver disease among patients with Hepatitis C Virus: a large state Medicaid perspective

**DOI:** 10.1186/1472-6963-12-459

**Published:** 2012-12-15

**Authors:** Joseph Menzin, Leigh Ann White, Christine Nichols, Baris Deniz

**Affiliations:** 1 Boston Health Economics, Inc, 20 Fox Road, Waltham, MA, 02451, USA; 2 Vertex Pharmaceuticals Incorporated, 130 Waverly Street, Cambridge, MA, 02139, USA

**Keywords:** Hepatocellular carcinoma, Decompensated cirrhosis, Cost, Administrative claims databases, Payers, HCV, Hepatitis C

## Abstract

**Background:**

Chronic hepatitis C virus (HCV) may progress to advanced liver disease (ALD), including decompensated cirrhosis and/or hepatocellular carcinoma (HCC). ALD can lead to significant clinical and economic consequences, including liver transplantation. This study evaluated the health care costs associated with ALD among HCV infected patients in a Medicaid population.

**Methods:**

Using Florida Medicaid claims data, cases were patients with at least 1 diagnosis of HCV or prescription therapy for HCV (ribavirin plus interferon, peginterferon, or interferon alfacon-1) prior to an incident ALD-related diagnosis (“index event”) between 1999 and 2007. ALD-related conditions included decompensated cirrhosis, HCC, or liver transplant. A cohort of HCV patients without ALD (comparison group subjects) were matched 1-to-1 based on age, sex, and race. Baseline and follow-up were the 12 months prior to and following index, respectively; with both periods allowing for a maximum one month gap in eligibility. For both case and comparison patient cohorts, per-patient-per-eligible month (PPPM) costs were calculated as total Medicaid paid amount for each patient over their observed number of eligible months in follow-up, divided by the patient’s total number of eligible months. A generalized linear model (GLM) was constructed controlling for age, race, Charlson score, alcoholic cirrhosis, and hepatitis B to explore all-cause PPPM costs between study groups. The final study group included 1,193 cases and matched comparison patients (mean age: 49 years; 45% female; 54% white, 23% black, 23% other).

**Results:**

The majority of ALD-related diagnoses were for decompensated cirrhosis (92%), followed by HCC (6%) and liver transplant (2%). Cases had greater comorbidity (mean Charlson score: 3.1 vs. 2.3, *P* < 0.001). All-cause inpatient use up to 1-year following incident ALD diagnosis was significantly greater among cases with ALD (74% vs. 27%, *P* < 0.001). In the GLM, cases had 2.39 times greater total adjusted mean all-cause PPPM costs compared to the comparison group ($4,956 vs. $1,735 respectively; P < 0.001). Among cases, mean total unadjusted ALD-related costs were $1,356 PPPM, which were largely driven by inpatient costs ($1,272).

**Conclusions:**

Our results suggest that among patients diagnosed with HCV, the incremental costs of developing ALD are substantial, with inpatient stays as the main driver of these increased costs.

## Background

In 2008, the World Health Organization estimated that between 2.2 and 3.0% of the world’s population was infected with the hepatitis C virus (HCV), or approximately 130–170 million individuals [1]. In the United States, it is estimated that approximately 1.3% of the population is infected [2]. The majority of infections occurred over 20 years ago with the peak incidence of acute HCV infections estimated at 291,000 cases in 1989 and dropping to approximately 18,000 incident cases in 2008 [3]. This marked decrease in incidence is in part due to improved blood donor screening and safer injection drug practices. As most patients were infected two decades ago, there is a relatively low prevalence of HCV infection among patients younger than age 45 compared to older age groups, with peak prevalence in the US occurring among patients aged 40–49 years of age in 2010 [2].

The economic burden of HCV is substantial with annual total all-cause health care costs estimated between $9,576 and $22,424 (adjusted to 2009 dollars) [4-7] and HCV-related costs estimated at approximately $7,343 in the first year following HCV diagnosis (2009 dollars) [5]. The main source of clinical and patient burden stems from the onset of decompensated cirrhosis and progression to hepatocellular carcinoma (HCC). As liver disease caused by HCV progresses slowly, many patients with HCV remain asymptomatic for years following infection with the virus [8]. Results from a recent model (ignoring effects of HCV treatment) showed that as the duration of HCV infection increases in surviving cohorts today, the proportion of patients with cirrhosis and other liver complications, such as hepatic decompensation, HCC, and liver-related deaths will increase dramatically over the next 10 years [9]. The same model estimated that mortality from HCV-related liver disease increased by 150% between the 1990s and 2000s, from a total of 56,377 deaths between 1990–1999 to 145,667 deaths between 2000 and 2009 [9]. Currently, approximately one-third of HCC cases in the US are attributed to HCV-related liver damage and HCV is the leading cause of liver transplantation [10]. A recent US-based model estimated that as HCV progresses, the proportion of all HCV patients with cirrhosis would increase from 25% in 2010 to 37% in 2020 [9].

Although the clinical progression to advanced liver disease (ALD) among patients with HCV is documented, little is known about the economic burden of ALD in a Medicaid population. State Medicaid programs covered approximately 48.6 million persons in 2010, and is expected to grow substantially when the Patient Protection and Affordable Care Act is fully enacted over the next several years [11]. In one modeling study, Wong et al. projected that direct medical costs in the US of managing future HCV-related disease (i.e. ALD-related diagnoses) could range between $6.5 and $13.6 billion between the years 2010 and 2019 with substantial additional societal costs attributed to approximately 720,000 years of life lost due to HCV-related decompensated cirrhosis and HCC [12]. Additionally, a 2012 systematic literature review of the economic burden of HCV-associated disease concluded that most cost estimates were from 1990, and were in need of updating given the aging cohort of HCV-infected patients [13].

As the HCV population is aging with a growing proportion of patients developing ALD-related complications in the coming years [9,14], there is a need for research on the direct health care cost burden of HCV-related ALD. The goal of this study was to assess the resource use and costs associated with ALD among patients diagnosed with HCV using administrative claims data from a large state Medicaid program.

## Methods

### Overview

This retrospective database analysis used administrative claims and enrollment records from Florida Medicaid database over the period from July 1, 1998 through June 30, 2008. Study cases were identified based on medical claims with a diagnosis of HCV or a relevant drug therapy (see below for HCV therapies) with an incident ALD-related diagnosis; non-ALD matched comparison group patients were selected to estimate the incremental burden of ALD. Key study measures included all-cause and ALD-related resource use and costs by resource use component including inpatient, outpatient, pharmacy, and other resource use.

### Data source

The Florida Medicaid program covers approximately 3 million low-income or disabled individuals or approximately 16% of the Florida population in 2008 [15,16]. The Florida Medicaid database consists of 2 patient de-identified files: (1) a claims file, with a de-identified patient ID, details on medical and pharmacy utilization, including date of service, place of service, *International Classification of Diseases, Ninth Revision, Clinical Modification* (ICD-9-CM) codes, procedure codes, physician specialty, national drug codes, drug quantity dispensed, days supplied, and Medicaid paid amounts; and (2) an eligibility file, with de-identified patient ID, details on monthly enrollment and patient demographics, and date of death as of the latest year of available claims. No information on specific causes of death is available.

### Patient selection

#### Cases

Patients were initially identified as eligible cases if they had at least 1 HCV diagnosis including acute, chronic, and “other unspecified HCV” codes (ICD-9-CM codes: 070.41, 070.51, 070.44, 070.54, 070.70, 070.71, V02.62) in any of 5 diagnosis fields on an inpatient or outpatient claim, or prescription therapy indicative of HCV (at least one claim for ribavirin and at least one additional claim for either interferon, peginterferon, or interferon alfacon-1 within the same fiscal year). In addition, cases were required to have an incident ALD-related diagnosis or procedure, defined by medical claims for at least 1 of the following conditions (using ICD-9-CM codes) between July 1, 1999 and June 30, 2007, and no such diagnosis in the prior 12 months:

(1) Decompensated cirrhosis diagnosis: esophageal varices with/without bleeding (ICD-9-CM codes 456.0x, 456.1x), esophageal varices in diseases classified elsewhere with/without bleeding (456.20, 456.21), ascites (789.5x), hepatic coma/encephalopathy or unspecified encephalopathy (572.2x, 348.3x), hepatorenal syndrome (572.3x), portal hypertension (572.4x);

(2) HCC (155.0x); or

(3) Liver transplant procedure (50.51, 50.59; or CPT-4 codes 47135, 47136), or history of liver transplant diagnosis (V42.7).

The date of the first ALD-related diagnosis was termed the “index date” with the baseline period defined as the 12 months prior to the index date and the follow-up period defined as the 12 months following index (including the index date). Patients were allowed a maximum one month gap in Medicaid eligibility in the baseline and/or follow-up periods. Moreover, patients who died had their follow-up end as of the date of death. An HCV diagnosis was required in the baseline period prior to the first ALD-related diagnosis. If patients had more than 1 ALD-related diagnosis on their index date the following hierarchy was applied to create 3 mutually exclusive groups: liver transplant diagnosis or procedure code, followed by HCC diagnosis and decompensated cirrhosis diagnosis. If patients were enrolled in Medicare or a Health Maintenance Organization (HMO) at any time during the study period they were excluded from analyses since complete claims may not be available for them.

#### Comparison cases

A comparison group was matched 1-to-1 with cases based on 5-year age groups, sex, and race after they met the eligibility criteria: had at least 1 inpatient or outpatient diagnosis of HCV in any of 5 diagnosis fields on an inpatient or outpatient claim, or prescription therapy indicative of HCV; had no diagnosis of ALD in the baseline or follow-up period; was not enrolled in Medicare or an HMO at any time during the study period; and had continuous Medicaid eligibility in the entire baseline and follow-up periods (including an allowed maximum one month gap in Medicaid eligibility) or until death, whichever occurred first. Each eligible comparison patient was assigned the same index date as his/her matched case.

### Study measures

Study measures included baseline demographic and clinical characteristics, selected outcomes, and resource use and cost measures evaluated over the follow-up period. Patients’ age was assessed at the index date and Charlson comorbidities were assessed in the baseline period. The Deyo-Charlson comorbidity score is a measure of physical health status commonly used in studies of medical claims and chronic disease [17-19]. This score is calculated by assigning points to presence of the following 17 conditions (with higher scores indicating poorer overall health status): myocardial infarction, congestive heart failure, peripheral vascular disease, cerebrovascular disease, dementia, COPD, rheumatologic disease, peptic ulcer disease, mild liver disease, diabetes mellitus (uncomplicated), diabetes with chronic complications, hemiplegia or paraplegia, renal disease, any malignancy, liver disease, metastatic solid tumor, and AIDS. In this analysis, we excluded “liver disease” from the Charlson score calculation as this was a selection criterion for our “case” cohort.

Prevalence of other ALD and non-ALD related selected diagnoses (using ICD-9-CM codes) evaluated in the follow-up period including: alcoholic cirrhosis (571.1x), diabetes without chronic complications (250.0x-250.3x, 250.7x), gastrointestinal bleeding (578.xx, 530.82), hepatitis B virus (HBV) (070.2x, 070.3x), human immunodeficiency virus (042.xx), other sequelae of chronic liver disease (572.8x), acute renal failure (584.5x-584.9x), and unspecified disorder of the liver (573.9x). Prevalence of selected ALD-related diagnoses in follow-up were also reported (if their prevalence was >5%). Mortality was also assessed for both the case and comparison cohorts over follow-up. Both all-cause and ALD-related resource use and costs (defined as the Medicaid paid amount) were assessed in the follow-up period, including hospitalizations and hospital days, outpatient services, pharmacy prescriptions (analyzed for all-cause resource use and costs only), and other resource use (including nursing home stays, home health, hospice, and other services). ALD-related costs included any payments from claims with a primary or secondary ALD-related diagnosis or procedure, as listed above.

### Data analyses

Descriptive analyses of all study measures were performed. All-cause resource utilization and costs were reported both for the case and comparison group cohorts, while ALD-related resource use and costs were reported for cases only, stratified by index ALD type. This only included decompensated cirrhosis or HCC since the transplant group included only 21 patients of whom only one underwent an actual transplant procedure during the study period. Differences between cases and comparison group patients were tested for statistical significance (defined as *P* < 0.05) with t-tests used to evaluate the statistical significance of differences in continuous variables with approximately normal distributions, nonparametric rank-order Wilcoxon tests for continuous variables with skewed distributions, and Cochran-Mantel-Haenszel or Fisher’s exact tests used for comparisons of proportions. Resource use during follow-up was reported as the percentage of patients with use in each category of interest and mean units of resource use (e.g., hospital admissions, hospital days) were reported on a per-patient-per-eligible month (PPPM) basis.

Costs were defined as the Medicaid paid amount over follow-up, inflation-adjusted using the medical care component of the consumer price index to 2009 dollars. For both case and comparison group cohorts, PPPM costs were calculated as total Medicaid paid amount for each individual patient over their entire number of eligible months in follow-up, divided by the patient’s total number of eligible months, with summary statistics (mean, standard deviation, range) calculated for the entire cohort. A generalized linear model using a log-link function and negative binomial distribution was used to adjust total all-cause costs for potential confounding factors including age, race, Charlson score, comorbid alcoholic cirrhosis, and HBV. This model functional form was chosen since our cost data were significantly right-skewed). SAS software (Version 9.0, SAS Institute, Cary, NC) was used for all analyses.

## Results

### Demographic and clinical characteristics

**Figure 1:**
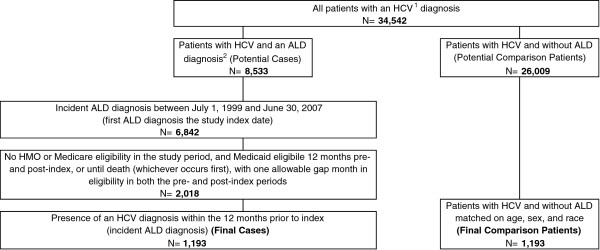
**Patient selection flowchart.** Source: Florida Medicaid database: claims with dates of service between July 1, 1998 and June 30, 2008. ^1^ Hepatitis C Virus (HCV) was defined as acute, chronic, and other unspecified HCV ^2^ Advanced Liver Disease (ALD) was defined as decompensated cirrhosis, hepatocellular carcinoma, or liver transplant procedure/history.

There were 34,542 patients identified with a diagnosis or prescription therapy indicative of HCV, of whom 8,533 had a diagnosis of both HCV and ALD, and 26,009 had a diagnosis of HCV alone. After applying all selection criteria 1,193 cases and 1,193 matched comparison group patients were available for analysis (Figure 1). The population was predominantly male (54.6%), and middle-aged (mean age 49 years), with just over half of patients identifying their race/ethnicity as White (54.1%) (Table 1). The baseline mean ± SD Charlson score (excluding HCC, mild liver disease, and liver disease) was significantly higher among cases (3.1 ± 3.2) than comparison group patients (2.3 ± 2.7) (*P* < .001), with the most common Charlson comorbidities among cases being chronic pulmonary disease (34.0%), diabetes (25.4%), and acquired immune deficiency syndrome (24.6%). Approximately one-third of cases (35.1%) and less than 1% of comparison group patients died during a mean (±SD) follow-up of 277 ± 135 days among cases and 366 ± 8 days among comparison group patients (Table 1).

**Table 1:** Baseline and follow-up demographic, clinical, and survival characteristics

	**Patients with HCV and ALD (Cases)**	**Patients with HCV and without ALD (Controls)**	***P-value***
N	1193	1193	
Baseline			
Age at index date			
Mean	48.6	49.0	0.268
SD	8.6	8.7	
Median	49.0	49.0	
<35	5.0%	4.4%	
35-44	22.8%	21.2%	
45-54	50.3%	50.6%	
55+	21.9%	23.8%	
Male (%)	54.6%	54.6%	1.000
Race			
White	54.1%	54.1%	1.000
Black	23.3%	23.3%	1.000
Hispanic	9.8%	9.8%	1.000
Other	12.8%	12.8%	1.000
Charlson score^1^			
Mean	3.1	2.3	< .001
SD	3.2	2.7	
Median	2.0	1.0	
Follow-Up			
Duration of follow-up (days)			
Mean	277	366	< .001
SD	135	8	
Median	366	366	
Survival			
Died during evaluation period (%)	35.1%	0.8%	< .001
Selected non-ALD-related diagnoses (%)			
Alcoholic cirrhosis	28.8%	1.1%	< .001
Diabetes	24.8%	19.8%	0.004
Gastrointestinal bleeding	20.2%	3.6%	< .001
HBV	9.7%	2.9%	< .001
HIV	25.2%	23.7%	0.418
Other sequelae of chronic liver disease	10.5%	0.3%	< .001
Acute Renal failure	13.1%	2.2%	< .001
Unspecified disorder of the liver	11.2%	1.8%	< .001
Selected ALD-related diagnoses (%)			
Ascites	54.5%	0.0%	< .001
Esophageal varices without bleeding	11.2%	0.0%	< .001
Hepatic coma (encephalopathy)	19.4%	0.0%	< .001
Portal hypertension	11.7%	0.0%	< .001

Source: Florida Medicaid database: claims with dates of service between July 1, 1998 and June 30, 2008.

Note: Comparison patients were matched based on 5-year age groupings, sex, and race to cases.

^1^T-tests were used to evaluate differences in mean age and Charlson score, while Wilcoxon tests were used for duration of follow-up. The Fisher Exact 2-tailed test was used for comparisons of proportions.

^2^Race/ethnicity was patient-identified; with "Other" race/ethnicity including American Indian, Asian, and others.

^3^Charlson score excludes HCC, 'Mild Liver Disease' and 'Liver Disease' comorbidities.

^4^Significance testing was not performed on ALD-related diagnoses as the comparison cohort could not have any of these diagnoses in baseline or follow-up by definition. These diagnoses were based on all inpatient and outpatient diagnoses observed over the follow-up period: ALD advanced liver disease*, HBV* hepatitis B virus, *HCV* hepatitis C virus, *HIV* human immunodeficiency virus.

Among cases, nearly all had decompensated cirrhosis as the index ALD-related claim (N = 1,098; 92.0%), followed by HCC (N = 73; 6.1%) or liver transplant (N = 22; 1.8%) claims. Four patients had both a decompensated cirrhosis and HCC claim on their index date and were thus considered to have HCC as their index ALD-related diagnosis. Among patients who were initially diagnosed with decompensated cirrhosis or HCC diagnosis at index, 3.1% and 6.8% received a liver transplant in follow-up, respectively. The most common ALD-related diagnoses among cases in follow-up were ascites (54.5%) and alcoholic cirrhosis (28.8%). Additionally, HIV (25.2%) and diabetes (24.8%) were prevalent diagnoses in follow-up (Table 1).

### Medical resource use

A significantly higher proportion of cases than comparison group patients had at least 1 all-cause inpatient hospitalization (73.6% vs. 27.1%), outpatient service use (99.2% vs. 96.1%), or other resource use (91.3% vs. 88.4%) in the follow-up period (all *P* < .001, except other resource use *P* = .025) (Table 2). The mean number of hospital admissions PPPM was significantly higher among cases than comparison group patients (0.3 vs. 0.1, *P* < .001), as were the mean number of hospital days PPPM (1.9 vs. 0.3, *P* < .001). Additionally, cases had 15.4 more outpatient claims PPPM compared to comparison group patients (19.4 vs 4.1; *P* < .001).

**Table 2:** All-cause health care resource use among patients with HCV and ALD (cases) and matched patients with HCV and no ALD (comparison)

	**Patients with HCV and ALD (Cases)**	**Patients with HCV and without ALD (Comparison)**	**Difference**	***P*-value**^**1**^
N	1,193	1,193		
Inpatient				
Percent with ≥1 hospitalization (1 yr follow-up)	73.6%	27.1%	46.5%	< .001
Hospital admissions (PPPM)				< .001
Mean	0.3	0.1	0.3	
SD	0.4	0.1		
Median	0.2	0.0		
Hospital days (PPPM)				< .001
Mean	1.9	0.3	1.6	
SD	2.9	0.8		
Median	0.7	0.0		
Outpatient Services^2^				
Percent with ≥1 outpatient service (1 yr follow-up)	99.2%	96.1%	3.1%	< .001
Outpatient office/clinic services (PPPM)				< .001
Mean	19.4	4.1	15.3	
SD	24.2	4.7		
Median	10.4	2.6		
Pharmacy				
Percent with ≥1 medication dispensed (1 yr follow-up)	89.8%	92.0%	−2.2%	0.064
Medications dispensed (PPPM)				0.010
Mean	5.8	5.4	0.4	
SD	4.5	4.3		
Median	5.2	4.6		
Other Services^3^				
Percent with at least 1 other resource use claim	91.3%	88.4%	2.9%	0.025

Source: Florida Medicaid database: claims with dates of service between July 1, 1998 and June 30, 2008.

Notes: Percentages are those with the resource use of interest at any time during the year.

^1^ Calculated with the Fisher Exact 2-tailed test used for comparison of proportions, and the Wilcoxon test for comparison of means.

^2^ Patients may have received multiple outpatient services on the same day, including hospital outpatient, physician, nurse practitioner, physician assistant, clinic, or other outpatient services.

^3^ "Other" services include nursing home, home health, hospice, and other services.

*ALD* advanced liver disease, *HCV* hepatitis C virus, *PPPM* per-patient-per-month.

Among cases, nearly half had an ALD-related inpatient hospitalization in one year of follow-up (43.8%), 89.0% had ALD-related outpatient service use, and 5.5% had ALD-related other service use (Table 3). Patients with a decompensated cirrhosis diagnosis on their index date were more likely to have ALD-related inpatient hospitalizations (45.0% vs. 37.0%) or ALD-related outpatient service use in follow-up (90.4% vs. 87.7%) versus those with an HCC diagnosis at index, although these differences were not statistically significant. Conversely, cases with an HCC claim at index had a significantly greater percentage with “other” service use (including nursing home, home health, hospice, and other services) during follow-up versus those with a decompensated cirrhosis claim (26.0% vs 4.2%, *P* < .001). The number of hospital admissions on a PPPM basis was similar for those with decompensated cirrhosis versus HCC as the index ALD type (0.1 vs 0.2 admissions PPPM, *P* = .445), as were mean number of hospital days (0.9 vs 1.4 days, *P* = 0.592).

**Table 3:** ALD-related health care resource use among patients with HCV and ALD (cases), by ALD type at index diagnosis

**Variables**	**Index diagnosis**			
	**Overall**^**1**^	**Decompensated cirrhosis**	**Hepatocellular carcinoma**	***P*-value**^**2**^
N	1193	1098	73	
Inpatient				
Percent with a hospitalization (1 yr follow-up)	43.8%	45.0%	37.0%	0.224
Hospital admissions (PPPM)				
Mean	0.1	0.1	0.2	0.445
SD	0.3	0.3	0.3	
Median	0.0	0.0	0.0	
Hospital days (PPPM)				
Mean	0.9	0.9	1.4	0.592
SD	2.1	2.0	3.2	
Median	0.0	0.0	0.0	
Outpatient Services^3^				
Percent with an outpatient service (1 yr follow-up)	89.0%	90.4%	87.7%	0.416
Outpatient office/clinic services (PPPM)				
Mean	1.8	1.7	4.8	0.251
SD	5.9	5.0	13.4	
Median	0.3	0.3	0.3	
Other Services^4^				
Percent with other resource use (1 yr follow-up)	5.5%	4.2%	26.0%	<.001

Source: Florida Medicaid database: claims with dates of service between July 1, 1998 and June 30, 2008.

Notes: Percentages are those with the resource use of interest at any time during the year. ALD-related resource use includes all claims with an ALD-related code.

1 “Overall” includes 22 patients with an index event defined by either a liver transplant procedure or a diagnosis code indicating history of a liver transplant.

^2^Calculated with the Fisher Exact 2-tailed test used for comparison of proportions, and the Wilcoxon test for comparison of means.

^3^Patients may have received multiple outpatient services on the same day, including hospital outpatient, physician, nurse practitioner, physician assistant, clinic, or other outpatient services.

^4^ "Other" services include nursing home, home health, hospice, and other services.

*ALD* advanced liver disease, *HCV* hepatitis C virus, *PPPM* per-patient-per-month.

### All-cause and ALD-related medical costs

The total unadjusted all-cause medical costs were estimated at $37,424 among cases and $20,762 among comparison patients in the entire follow-up period (not accounting for months of eligibility). Consistent with resource use findings, patients with HCV and ALD had significantly higher unadjusted inpatient, outpatient, and other costs versus patients with HCV and no ALD (all *P* < .001). The total incremental unadjusted PPPM cost of ALD among patients with HCV averaged $3,207, comprised of $2,312 in incremental inpatient costs, $498 in incremental outpatient service costs, and an additional $424 in other costs (Table 4). In a generalized linear model with negative binomial distribution that controlled for age, race, Charlson score, comorbid alcoholic cirrhosis, and HBV, cases had 2.39 times greater total adjusted mean all-cause PPPM costs compared to comparison group patients (Table 5) ($4,956 vs. $1,735 respectively; *P* < 0.001), which is slightly lower than our unadjusted estimate (2.85 or $4,937 / $1,730). Total ALD-related costs among cases averaged $1,356 PPPM, with patients having HCC as an index ALD diagnosis exhibiting greater costs versus those with decompensated cirrhosis as the index ALD diagnosis ($2,297 vs. $1,319, *P* = 0.807) (Table 6).

**Table 4:** All-cause PPPM healthcare costs among Medicaid beneficiaries with HCV and ALD (cases) and matched patients with HCV and no ALD (comparison)

	**Patients with HCV and ALD (Cases)**	**Patients with HCV and without ALD (Comparison)**	**Difference**	***P*-value**^**1**^
N	1193	1193		
Inpatient				
Mean (SD)	$2,649 ($4,179)	$337 ($981)	$2,312	< .001
Median	$905	$0		
IQR	$0-$3,430	$0-$79		
Outpatient^2^				
Mean (SD)	$713 ($824)	$215 ($320)	$498	< .001
Median	$467	$132		
IQR	$249-$902	$49-$270		
Pharmacy				
Mean (SD)	$791 ($1,401)	$818 ($1,312)	-$27	0.032
Median	$385	$462		
IQR	$126-$895	$132-$1,029		
Other costs^3^				
Mean (SD)	$784 ($1,541)	$360 ($1,265)	$424	< .001
Median	$171	$90		
IQR	$51-$669	$18-$208		
Total costs				
Mean (SD)	$4,937 ($5,236)	$1,730 ($2,309)	$3,207	< .001
Median	$3,146	$1,002		

Source: Florida Medicaid database: claims with dates of service between July 1, 1998 and June 30, 2008.

Notes: All costs are inflated to 2009 USD using the Medical Care component of the CPI. Costs over the entire follow-up period were divided by the number of eligible patient months to create the PPPM cost estimates.

^1^Calculated with the Wilcoxon test for comparison of means.

^2^Patients may have received multiple outpatient services on the same day, including hospital outpatient, physician, nurse practitioner, physician assistant, clinic, or other outpatient services.

^3^ "Other" costs include nursing home, home health, hospice, and other services.

*ALD* advanced liver disease, *HCV* hepatitis C virus, *PPPM* per-patient-per-month.

**Table 5:** **Generalized linear model**^**1**^**predicting adjusted all-cause PPPM healthcare costs**

**Parameter**	**Beta-coefficient**	**Odds ratio**	***P*-Value**
Male	0.0508	1.05	0.266
Charlson Score^2^ of 1	0.2977	**1.35**	**<.001**
Charlson Score of 2	0.4745	**1.61**	**<.001**
Charlson Score ≥3	0.8231	**2.28**	**<.001**
Alcoholic Cirrhosis	0.2759	**1.32**	**<.001**
Hepatitis B Virus (HBV)	0.3638	**1.44**	**<.001**
Age (Continuous Variable)	−0.0048	1.00	0.061
Black	0.1557	**1.17**	**0.007**
Hispanic	−0.1095	0.90	0.169
Other Race	0.0869	1.09	0.219
Cases (HCV with ALD)	0.8728	**2.39**	**<.001**

Source: Florida Medicaid database: claims with dates of service between July 1, 1998 and June 30, 2008.

Reference groups included: Females, Charlson score 0, patients with no diagnosis of alcoholic cirrhosis or HBV, Whites, and comparison patients (HCV with no ALD).

Model fit statistics: Degrees of freedom (2374), scaled deviance (2842), log-likelihood (61454257).

1 Constructed using a using a log-link function and negative binomial distribution.

^2^ Charlson score excludes HCC, 'Mild Liver Disease' and 'Liver Disease' comorbidities.

*PPPM* per-patient-per-month.

**Table 6:** ALD-related PPPM healthcare costs among Medicaid beneficiaries with HCV and ALD (cases), by ALD type at index ALD diagnosis

		**Index diagnosis**		
**Variables**	**All ALD-related costs**^**1**^	**Decompensated cirrhosis**	**Hepatocellular carcinoma**	***P*-value**^**2**^
N	1193	1098	73	
Inpatient				
Mean (SD)	$1,272 ($2,926)	$1,242 ($2,806)	$2,093 ($4,539)	0.624
Median	$0	$0	$0	
IQR	$0-$1,006	$0-$1,056	$0-$1,155	
Outpatient				
Mean (SD)	$83 ($184)	$76 ($168)	$202 ($341)	0.051
Median	$23	$23	$37	
IQR	$6-$79	$7-$78	$8-$157	
Other costs^3^				
Mean (SD)	$1 ($11)	$1 ($11)	$2 ($10)	<.001
Median	$0	$0	$0	
IQR	$0-$0	$0-$0	$0-$0	
Total costs^4^				
Mean (SD)	$1,356 ($2,981)	$1,319 ($2,848)	$2,297 ($4,700)	0.807
Median	$88	$96	$67	

Source: Florida Medicaid database: claims with dates of service between July 1, 1998 and June 30, 2008.

Notes: All costs are inflated to 2009 USD using the Medical Care component of the CPI. Costs over the entire follow-up period were divided by the number of eligible patient months to create the PPPM cost estimates.

^1^ “ALD-Related Costs” includes all patients with HCV and ALD, including 22 patients whose index ALD diagnosis was a transplant procedure or history of transplant.

^2^ Calculated with the Wilcoxon test for comparison of means.

^3^ "Other" costs include nursing home, home health, hospice, and other services.

^4^ "Total costs" include inpatient, outpatient, pharmacy, and other (nursing home, home health, hospice, and other services) costs. Pharmacy and other costs comprised < $5 of mean total ALD-related costs in each cohort.

*ALD* advanced liver disease, *HCV* hepatitis C virus, *PPPM* per-patient-per-month.

## Discussion

In this study using data from the Florida Medicaid program, the fourth-largest Medicaid program in the United States, we found that patients with HCV with an incident ALD-related diagnosis (cases) had significantly greater all-cause resource use and costs compared to patients with HCV without an ALD diagnosis (comparison patients). Adjusted total mean all-cause costs PPPM in the year following the first ALD-related diagnosis among cases were estimated at $4,956, while mean total PPPM costs for comparison patients were estimated at $1,735. A substantially larger proportion of all-cause costs were comprised of inpatient hospitalizations among cases (54%) vs. controls (19%). Total ALD-related costs in the year following an incident ALD-related diagnosis among patients with both HCV and ALD averaged $1,356 PPPM, or approximately 27% of all-cause costs in this cohort. These increased costs among patients with HCV and ALD may be linked to the observed higher use of healthcare resources in this cohort as shown in these analyses.

There are limited data on the incremental cost burden of ALD among Medicaid patients with HCV. Past research has focused primarily on the burden of HCV in general, not specific to patients with and without ALD-related diagnoses. Additionally, prior research has focused on patients covered by commercial health plans [4-7,20], which may be different than those covered by a state Medicaid program. Despite these differences, previous studies showed that annual health care costs among patients with HCV ranged from $9,576 to $22,424 (adjusted to 2009 USD) [4-7], which is similar to the all-cause estimate among our comparison group of patients with HCV and no ALD ($20,762). In an analysis of patients with an incident diagnosis of chronic HCV who were enrolled in a managed care organization (MCO), the annual all-cause costs in the first year following diagnosis were estimated at $22,424 for patients with HCV and $5,831 for patients without HCV (incremental $16,593; adjusted to 2009 USD) [5]. In a similar analysis of private MCO data, Poret et al. estimated total all-cause average payments in the year following the first HCV diagnosis at approximately $17,491 versus $3,020 in a comparison cohort with no HCV (adjusted to 2009 USD) [6]. Armstrong and colleagues reported total annual all-cause costs among patients with HCV who were treated with interferon alfa plus ribavirin therapy and enrolled in a large MCO to be approximately $9,577 (adjusted to 2009 USD) [4]. Of the total all-cause costs among treated patients with HCV, approximately $8,929 were HCV-related (adjusted to 2009 USD) [4]. In another analysis of MCO data, this one among patients with HCV aged less than age 65, Rosenberg et al. found total all-cause 3-year costs of $63,055 (annualized approximately $21,018; adjusted to 2009 USD) [7]. Finally, in a recent retrospective database analysis of administrative claims data from a large MCO the annualized total mean all-cause healthcare charges (not costs) per patient per year (PPPY) were higher for patients with diagnosis of HCV and ALD (decompensated cirrhosis: $27,000; HCC: $58,529; liver transplant: $113,116) compared to patients with HCV and no advanced liver disease ($14,917) (all in 2009 USD) [20]. These results are similar to our findings of higher costs among patients with HCV and ALD compared to HCV alone particularly among patients with diagnosis of HCC or receipt of a liver transplant, although data on charges are not directly comparable to the Medicaid expenditure information reported in our paper.

Although the incidence of HCV has declined substantially in the last decade, mortality due to liver-related complications is projected to increase over the next 25 years due to the aging of the prevalent HCV population. As the population ages, more patients will progress to development of decompensated cirrhosis or HCC. Increased ALD-related mortality and rates of progression will lead to a significant economic burden among the HCV-infected population. In one model-based estimate of the effect of treatment on HCV progression and liver-related mortality, increasing treatment rates among patients diagnosed with HCV from approximately 25% to 50% was projected to decrease the percentage of patients developing liver failure by 39%, HCC by 30%, and decrease the number of liver-related deaths by 34% between 2010 and 2019 [9].

The mortality rate observed among patients with diagnosis of HCV and ALD in this study was 35.1% over a mean of 277 days of follow-up. This estimate is in line with mortality rates reported in a systematic literature review of patients with cirrhosis [21]. Patients with stage 3 decompensated cirrhosis (i.e., patients with ascites and varices) were found to have a one-year mortality rate of 20% while those with stage 4 decompensated cirrhosis (i.e., those with GI bleeding with or without ascites) had a mortality rate of 57% at one year. Since over 90% of our patients with ALD had a diagnosis of decompensated cirrhosis, our population appears to be similar to the stage 3 and 4 cirrhosis patients examined in the review.

### Limitations

This study is subject to certain limitations that are common to all studies that rely on retrospective claims data, such as potential coding errors and incomplete data [22]. Our estimates of ALD-related resource use and costs may be slight overestimates, as we defined a claim with any ALD-related diagnosis (primary or secondary diagnosis) as disease-related, which may have in some cases captured nondisease related costs. Additionally, descriptive data also showed patients with HCV and ALD to have higher prevalence of other non-liver related comorbidities, therefore also potentially causing the incremental cost of ALD to be an overestimate. Although we controlled for both alcoholic cirrhosis and hepatitis B, two important comorbid conditions not included in the Charlson score, we cannot rule our residual confounding that could have led to an overestimate of excess costs. Finally, the difference in pharmacy costs observed (higher pharmacy costs among controls) may have skewed differences in total all cause costs between cases and controls due to a higher prevalence of contraindications to interferon-based treatment among patients with HCV and ALD. However, many of these patients were living with HCV for a number of years prior to the first year of our data (1998), and already presumably completed their course of treatment, thus not overly influencing pharmacy costs. However, we do acknowledge that the prevalence of treatment is likely higher in the control group and therefore influencing the significant difference in pharmacy costs observed in our dataset.

The main decrease in sample size in our study was due to the requirement that patients be Medicaid-only eligible with no HMO or Medicare coverage. Therefore, although this study was conducted using a large state Medicaid program, costs may not be generalizable to other Medicaid programs or to other payers. Additionally, exclusion of patients who are dually eligible for Medicaid and Medicare may bias cost estimates downward as Medicaid patients with Medicare are likely to be sicker than other patients [23].

## Conclusion

This retrospective database analysis showed a substantial incremental burden of ALD among patients with HCV enrolled in a large state Medicaid program. Patients with both HCV and ALD had an average of approximately $3,200 more in all-cause medical costs on PPPM basis compared to those with an HCV diagnosis alone. To our knowledge, this study is the first publication to assess the incremental cost of ALD among patients with HCV in a Medicaid population. There is a need for further research in this area, potentially among different patient populations (e.g. privately insured patients) or subpopulations (e.g., in HCV patients with limited access to treatment, patients co-infected with human immunodeficiency virus and/or hepatitis B virus). Additionally, studies of lifetime costs among patients with HCV progressing to ALD would be informative.

## Abbreviations

ALD: Advanced Liver Disease; HCC: Hepatocellular Carcinoma; HCV: Hepatitis C Virus; HMO: Health Maintenance Organization; MCO: Managed Care Organization; PPPM: Per-Patient-Per-Month; PPPY: Per-Patient-Per-Year.

## Competing interests

This research was performed by Boston Health Economics, Inc. and funded by Vertex Pharmaceuticals Incorporated.

## Authors’ contributions

JM, LAW, CN, and BD contributed to the concept and design of this study; CN contributed to programming; JM, LAW, CN, and BD contributed to evaluation and interpretation of results; and JM, LAW, CN, and BD contributed to manuscript preparation. All authors read and approved the final manuscript.

## Authors’ information

JM, and CN: Boston Health Economics, Inc., 20 Fox Road, Waltham, MA 02451.

## Pre-publication history

The pre-publication history for this paper can be accessed here:

http://www.biomedcentral.com/1472-6963/12/459/prepub
